# MicroRNA-124a Protects the Myocardium Against Ischemia Reperfusion Injury Through Regulation of the Notch Signaling Pathway

**DOI:** 10.21470/1678-9741-2020-0357

**Published:** 2022

**Authors:** Weijun Xu, Shan Jiang, Qi Liu

**Affiliations:** 1 Department of Emergency, Qingdao Hospital of Traditional Chinese Medicine (Qingdao Hiser Hospital), Qingdao, Shandong, People’s Republic of China.; 2 Department of Clinical Laboratory, Qingdao Chest Hospital, Qingdao, Shandong, People’s Republic of China.; 3 Department of Pulmonary Medicine Center, Qingdao Hospital of Traditional Chinese Medicine (Qingdao Hiser Hospital), Qingdao, Shandong, People’s Republic of China.

**Keywords:** MicroRNAs. Staining and Labeling. Myocardial Infarctation. Reperfusion. Tumor Necrosis Factor-alpha. Down-Regulation. Rats, Sprague-Dawley

## Abstract

**Introduction:**

This study’s objective is to investigate the effect of downregulation of micro ribonucleic acid (miR)-124a on myocardial injury after ischemia reperfusion (I/R) in rats.

**Methods:**

Sprague Dawley (SD) rats (n=20) were divided into four groups - sham, I/R, I/R+miR-124a antagomir (I/R+ant-miR-124a), and I/R+ant-normal control (NC). The pathomorphological and infarct size variance of injured myocardial tissues with IR were conducted with hematoxylin (HE) and triphenyltetrazolium chloride (TTC) staining. The expression levels of miR-124a, BAX, nuclear factor kappa B (NF-KB), Notch1, and Hes1 were examined by quantitative real-time polymerase chain reaction or Western blot in myocardium. The inflammatory cytokines interleukin (IL)-6, IL-1β, and tumor necrosis factor alpha (TNF-α) were detected by the enzyme-linked immunosorbent assay, as well as the activity of lactate dehydrogenase (LDH) and creatine kinase (CK) in serum by colorimetry.

**Results:**

The expression of miR-124a was increased in the I/R group. Compared with I/R and I/R+ant-NC groups, after downregulating miR-124a, the expression of IL-6, IL-1β, TNF-α, BAX, NF-KB, LDH, and CK were decreased, but the expression of Notch1 and Hes1 were increased. In HE staining, myocardial tissue edema, red blood cell exudation, and myocardial fiber arrangement disorder were accompanied by inflammatory cell infiltration and local necrosis in the I/R group. However, the pathological injury of myocardial tissue was alleviated after downregulating miR-124a. Additionally, TTC results showed that the myocardial infarction area was decreased in the I/R+ant-miR-124a group.

**Conclusion:**

Downregulation of miR-124a expression through Notch pathway can significantly reduce myocardial damage after 24 hours of I/R in SD rats. Therefore, miR-124a may become a potential therapeutic target for I/R injury.

**Table:** 

Abbreviations, acronyms & symbols				
ant	= Antagomir		NC	= Normal control
CK	= Creatine kinase		NF-κB	= Nuclear factor kappa B
ECG	= Electrocardiography		PPAR	= Peroxisome proliferator-activated receptor
ELISA	= Enzyme-linked immunosorbent assay		PVDF	= Polyvinylidene fluoride
GAPDH	= Glyceraldehyde 3-phosphate dehydrogenase		qRT-PCR	= Quantitative real-time polymerase chain reaction
HE	= Hematoxylin		RNA	= Ribonucleic acid
IL	= Interleukin		SD	= Sprague Dawley
I/R	= Ischemia reperfusion		TBST	= Tris-buffered saline with Tween 20
LDH	= Lactate dehydrogenase		TNF-α	= Tumor necrosis factor alpha
miR	= Micro ribonucleic acid		TTC	= Triphenyltetrazolium chloride
mRNA	= Messenger ribonucleic acid		WB	= Western blot

## INTRODUCTION

Cardiovascular disease, especially coronary artery disease, is the main cause of high morbidity and mortality in the world. Currently, there is no particularly effective treatment to protect the heart from myocardial ischemia reperfusion (I/R). Therefore, it is necessary to discover or develop new strategies to prevent myocardial I/R injury, so as to improve the clinical manifestations of patients with coronary heart disease ^[^^1^^]^. Myocardial I/R injury is mainly caused by cell death, tissue scar formation, and myocardial remodeling due to excessive formation of free radicals and Ca^2+^ overload. Recent studies have shown that Notch1 is the main receptor of Notch signaling pathway and is involved in a variety of anoxic states, including myocardial injury ^[^^2^^]^. Once ligands normally located on the surface of adjacent cells were activated, the receptor transmits the signal to the nucleus through intracellular proteolysis and intracellular domain release ^[^^3^^]^. This event caused the transcriptional activation complex to form and cause transcription of downstream target genes of Notch, such as Hes1 ^[^^4^^]^.

Micro ribonucleic acid (miR) is a single-stranded small molecule of ribonucleic acid (RNA) with a size of about 19-25 bases, which has negative regulation on gene expression. MiR, a post-transcriptional regulator of gene expression, regulates gene expression mainly by blocking protein translation or inducing degradation of messenger RNA (mRNA) ^[^^5^^]^. MiR regulates at least 60% of protein coding genes. MiR is a kind of highly stable molecular regulatory element widely existing in human tissues and body fluids, and changes in miR expression profiles can lead to many pathological processes. The first miR was discovered in 1993 in *Caenorhabditis elegans*, namely lin-4. Later, it was found that the second miR was let-7 ^[^^6^^]^. Alteration or abnormal expression of miR in human body can lead to various diseases, such as neurological genetic, immune, metabolic, and cardiovascular diseases. Meanwhile, miRs in blood and tissues are used as tumor markers for clinical tumor detection ^[^^7^^]^.

In recent years, studies have shown that miRs are involved in several cardiac-related processes including cardiac development, myocardial hypertrophy, heart failure, and angiogenesis. In the meantime, it has been found that under the condition of myocardial ischemia, the expression pattern of some miR in myocardial tissue has changed, and miR may participate in the effect of myocardial I/R on cardiac injury. Therefore, miR can be regulated to treat or relieve myocardial I/R injury to the heart.

Also, studies have shown that miR-124a can regulate acute chronic liver failure by negatively regulating glucocorticoid receptors ^[^^8^^]^ and can alleviate the onset of non-alcoholic fatty liver by being inhibited ^[^^9^^]^. Many studies have shown that miR-124a is related to tumor inhibition. For example, human glioma miR-124a inhibits the proliferation of malignant cells by inhibiting PHD finger protein 19 overexpression and enhancer of zeste homolog 2 overactivation ^[^^10^^]^. In addition, miR-124a has certain inhibitory effects on non-small lung cancer cells, liver cancer, uveal melanoma, gastric cancer, colorectal cancer and polyps, and medulloblastoma. Studies have found that miR-124a is highly expressed in the islets of type 2 diabetics and negatively regulates insulin secretion ^[^^11^^]^. We also found that miR-124a can regulate lipopolysaccharide-induced septic cardiac dysfunction by targeting STX2 ^[^^12^^]^.

However, the effect of miR-124a on myocardial injury after I/R has not been reported so far. Therefore, the purpose of this study is to explore the effect of miR-124a on myocardial injury after I/R in rats, so as to provide a new therapeutic target for myocardial I/R.

## METHODS

### Establishment of Rat Model of I/R

This study was approved by the Ethics Committee of Qingdao Hospital of Traditional Chinese Medicine (Qingdao Hiser Hospital). Twenty healthy adult male Sprague Dawley (SD) rats (180~220g, six weeks, specific-pathogen-free level) were randomly divided into four groups: sham operation group (Sham, n=5), I/R group (I/R, n=5), miR-124a antagomir group (I/R+ant-miR-124a, n=5), and miR-124a ant normal control group (I/R+ant-NC, n=5). SD rats were anesthetized by intraperitoneal injection of pentobarbital sodium (50 mg/kg). The SD rats’ limbs were fixed, their heart was fully exposed under cold light source, and the left descending anterior branch of the coronary artery was rapidly ligated for 30 min. At this time, the ST segment of the electrocardiography (ECG) could be seen to be increased, indicating the ligation was successful ^[^^13^^]^. After 30 minutes of ischemia, the relaxed ligation line of the rats showed that the elevated ST segment of ECG decreased and returned to normal level. After two hours of perfusion, the samples were collected, and the I/R modeling of SD rats was basically successful. After thoracotomy, the Sham group was directly sutured without ligation, and the remaining operations were the same as those of the I/R group. MiR-124a group was injected with miR-124a antagomir (0.2 µL/g) (Shanghai GenePharma Co., Ltd.) into the myocardium 24 hours before operation, and the treatment after 24 hours was the same as that of I/R group. I/R+ant-NC group was administrated with NC antagomir (0.2µL/g) (Shanghai GenePharma Co., Ltd.) within 24 hours of operation, and the treatment after 24 hours was the same as that of I/R group.

###  

### Hematoxylin (HE) Staining

After fixation with 10% neutral formaldehyde solution for 24 hours, the rat cardiac tissue in each group was routinely dehydrated, transparent, wax-soaked, and embedded, and coronal cut was performed (4 µm/tablet). The sections were dewaxed with xylene, rehydrated with ethanol, and stained with HE. The pathological changes were observed under optical microscope.

### Triphenyltetrazolium chloride (TTC) Staining

After reperfusion, the rat myocardial tissue was left to rest at -20℃ for 30 minutes, and the left ventricle was crosscut into 1 mm slices and placed in 1% TTC staining solution at 37℃ for 30 minutes. Cardiac muscle slices were immobilized overnight in 4% formalin solution. The unstained part of the myocardium was the infarction area, and the red area was the risk area of ischemia, which are weighed separately.

Colorimetry to Determine the Activities of Lactate Dehydrogenase (LDH) and Creatine Kinase (CK) in Serum

The blood samples were centrifuged at 4℃ for 15 minutes at 1000 r/min. The upper serum was absorbed and stored at -80℃ for subsequent tests. LDH in serum was evaluated with LDH cytotoxicity test kit (Beyotime Biotechnology Co., Ltd. Article No.: C0016), CK activity was evaluated with CK test kit (Nanjing Jiancheng Bioengineering Institute Article No.: A032) according to the instructions.

### Enzyme-Linked Immunosorbent Assay (ELISA)

Serum levels of the inflammatory factors tumor necrosis factor alpha (TNF-α), interleukin (IL)-6, and IL-1β were detected by ELISA. The protein samples and the standard were added to the well plate at 4℃ overnight, and the washing solution was washed off three times. The water was drained after each washing. Assay Diluent was added to each well and incubated at room temperature for two hours before washing. After washing with diluted primary antibody in each well, secondary antibody was added to the samples and those were incubated at room temperature for one hour and washed. Finally, after adding the substrate solution and the termination solution, the absorbance value was measured immediately at 450 nm in the enzyme marker, and the content of the substance to be measured was calculated by substituting it into the standard curve.

### Quantitative Real-time Polymerase Chain Reaction (qRT-PCR)

Two hours after reperfusion, SD rats' hearts were immediately taken and cleaned in phosphate-buffered saline, and some tissues were frozen in liquid nitrogen. Some myocardial tissue was added with TRIzol reagent (Invitrogen, Carlsbad, California, United States of America) to extract total mRNA from the cells. Subsequently, the extracted mRNA was reverse transcribed into complementary deoxyribonucleic acid under the following conditions: 25°C, 10 minutes, 50°C, 30 minutes, and 85°C, 5 minutes. Then qRT-PCR was performed using a fluorescent qRT-PCR kit (TaKaRa). In this study, primers of Primer5 designed by software were synthesized by Shanghai Sangon Biotech Co., Ltd. The primers were as follows: mRNA expression levels of miR-124a

The specific qRT-PCR conditions were: 95°C, 5 minutes, 95°C, 15 seconds, 60°C, 1 minute, a total of 40 cycles. The temperature in the melting curve was set to 60-95°C. All the experimental samples were set with three multiple wells and calculated and analyzed by 2^-△△Ct^ method ^[^^14^^]^.

### Western Blot (WB)

The protein expression levels of Notch1, Hes1, BAX, and nuclear factor kappa B (NF-κB) in rat cardiac tissue were detected by WB. An appropriate amount of radioimmunoprecipitation assay lysate was added to the myocardial tissue, and the protein lysate was obtained by homogenization centrifugation and stored in the refrigerator at -80 ℃. The protein samples were separated by 10% sodium dodecyl sulphate-polyacrylamide gel electrophoresis and transferred to the polyvinylidene fluoride (PVDF) membrane. The PVDF membrane was incubated with 5% skim milk at room temperature for one hour, and then the primary antibody was incubated in the refrigerator at 4℃ (1:1000, abcam) overnight. The film was washed with tris-buffered saline with Tween 20 (TBST) solution three times for 15 minutes. After the PVDF membrane was incubated at room temperature for one hour, the secondary antibody was removed and washed three times with TBST solution, each time for five minutes. After development, the protein bands were analyzed and calculated by Image J software, and glyceraldehyde 3-phosphate dehydrogenase was used as the internal reference standard.

### Statistical Analysis

The IBM Corp. Released 2011, IBM SPSS Statistics for Windows, Version 20.0, Armonk, NY: IBM Corp software was used to analyze the data. The data were tested for normality by probability-probability and quantile-quantile plots, which was found to be in accordance with normality, and further statistical analysis was carried out. The data were all expressed by mean ± standard deviation. The average number of each group was compared by one-way analysis of variance test and least significant difference-t method. P<0.05 indicates that the difference was statistically significant.

## RESULTS

### HE Staining

**Fig. 1:**
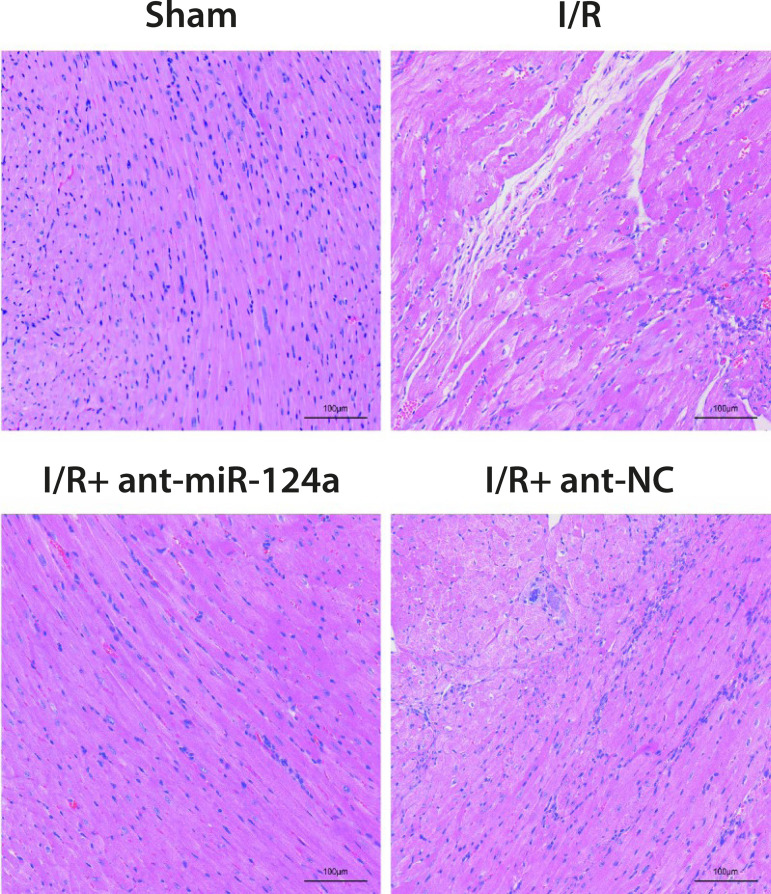
The effect of micro ribonucleic acid (miR)-124a on hematoxylin staining of myocardial tissue after myocardial ischemia reperfusion (I/R) in rats. ant=antagomir; NC=normal control

HE staining showed that the myocardial tissue morphology of the control group was basically normal, the myocardial fibers were arranged orderly, and there was no inflammatory cell infiltration in the stroma. Myocardial edema, red blood cell exudation, myocardial fiber arrangement disorder, accompanied by inflammatory cell infiltration and local necrosis, were observed in both I/R group and I/R+ant-NC group. Compared with I/R group and I/R+ant-NC group, these pathological features in I/R+ant-miR-124a group are relieved (Figure 1).

### TTC Staining

TTC staining results showed that 24 hours after I/R, myocardial tissue in I/R group and I/R+ant-NC group showed large-area infarction, with infarction areas of 42.32±3.58% and 39.56±3.22%, respectively, significantly increased compared with 5.82±2.78% in the control group (*P*<0.01); the infarct size in myocardial tissue of I/R+ant-miR-124a group was 28.85±2.98%, which was significantly lower than that of I/R group and I/R+ant-NC group (*P*<0.05). This indicated that upregulation of miR-124a expression significantly reduced the infarct area of the rats’ local cardiac tissue.

### Activity of LDH and CK in Serum

**Fig. 2:**
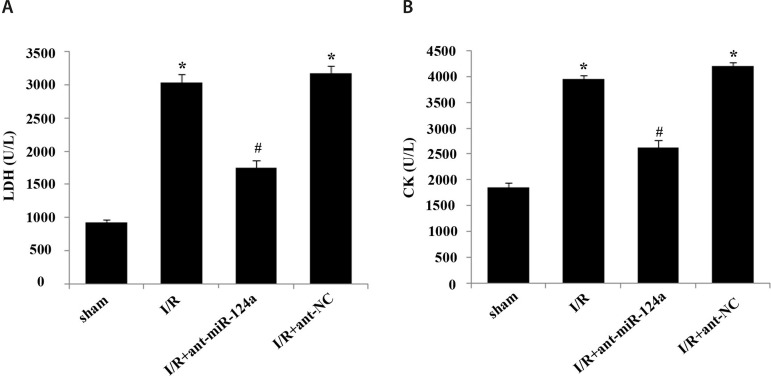
Effects of micro ribonucleic acid (miR)-124a on serum activities of lactate dehydrogenase (LDH) and creatine kinase (CK) in rats after ischemia reperfusion (I/R) injury. (A) Serum LDH activity in each group. (B) Serum CK activity in each group. Compared with sham group, *P<0.05; compared with I/R group, #P<0.05. ant=antagomir; NC=normal control

The activity test results of LDH and CK showed that compared with the control group, the levels of LDH (Figure 2A) and CK (Figure 2B) in serum of the I/R group and I/R+ant-NC group increased (*P*<0.05). Compared with the I/R group and I/R+ant-NC group, the levels of LDH (Figure 2A) and CK (Figure 2B) in serum of the I/R+ant-miR-124a group were significantly decreased (*P*<0.05).

### ELISA to Detect the Related Components in Serum

**Fig. 3:**
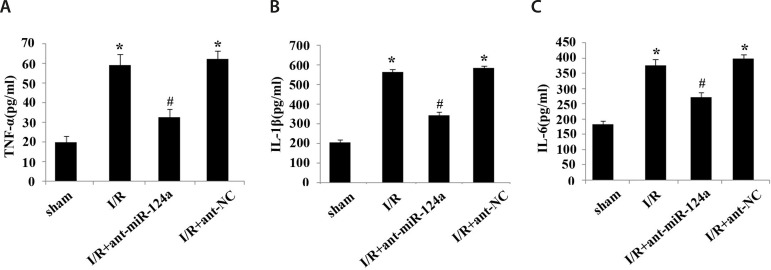
Concentration of inflammatory cytokines in cardiac tissue of ischemia reperfusion (I/R) rats. (A) Concentration of tumor necrosis factor alpha (TNF-α) in each group. (B) Concentration of interleukin (IL)-1β in each group. (C) Concentration of IL-6 in each group. Compared with sham group, *P<0.05; compared with I/R group, #P<0.05. ant=antagomir; miR=micro ribonucleic acid; NC=normal control

ELISA showed that compared with the control group, the levels of TNF-α (Figure 3A), IL-1β (Figure 3B), and IL-6 (Figure 3C) in serum in the I/R group and I/R+ ant-NC group were significantly increased. Compared with the I/R group and I/R+ant-NC group, the levels of TNF-α (Figure 3A), IL-6 (Figure 3B), IL-1β (Figure 3C) in serum in the I/R+ant-miR-124a group were significantly reduced (*P*<0.05).

### Related mRNA and Protein Expression Levels in Myocardial Tissues

**Fig. 4:**
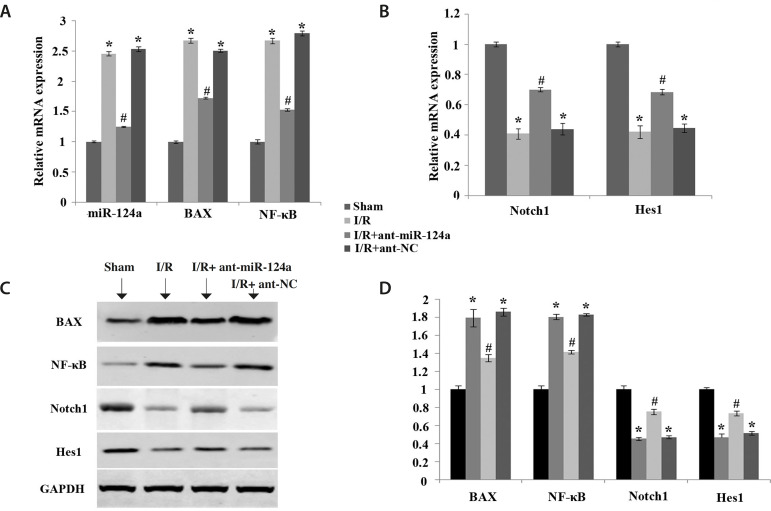
Effects of micro ribonucleic acid (miR)-124a on messenger ribonucleic acid (mRNA) and protein expression levels of related factors in the Notch pathway in rat myocardial tissue after ischemia reperfusion (I/R). (A) mRNA expression levels of miR-124a, BAX, and nuclear factor kappa B (NF-κB) in each group. (B) mRNA expression levels of Notch1 and Hes1 in each group. (C) Western blot was used to detect the protein expression levels of BAX, NF-κB, Notch1, and Hes1 in the myocardial tissues of each group. (D) Protein expression levels of BAX, NF-κB, Notch1, and Hes1 in the myocardial tissues of each group. The values are expressed as X±S. Compared with sham group, *P<0.05; compared with I/R group, #P<0.05. ant=antagomir; GAPDH=glyceraldehyde 3-phosphate dehydrogenase; NC=normal control

The results of qRT-PCR showed that compared with the control group, the mRNA expressions of miR-124a, BAX, and NF-κB in the I/R group and I/R+ant-NC group were significantly increased (*P*<0.05) (Figure 4A), and the mRNA expressions of Notch1 and Hes1 were significantly decreased (*P*<0.05) (Figure 4B). Compared with the I/R group and I/R+ant-NC group, the mRNA expressions of miR-124a, BAX, and NF-κB in the I/R+ant-miR-124a group were decreased (Figure 4A), while the mRNA expressions of Notch1 and Hes1 were increased (*P*<0.05) (Figure 4B).

WB detection results showed that compared with the control group, the protein expressions of BAX and NF-κB in the I/R group and I/R+ant-NC group were significantly increased (*P*<0.05), and the mRNA expressions of Notch1 and Hes1 were significantly decreased (*P*<0.05). Compared with the I/R group and I/R+ant-NC group, the mRNA expressions of BAX and NF-κB in the I/R+ant-miR-124a group were decreased, and the protein expressions of Notch1 and Hes1 were increased (*P*<0.05) (Figures 4C and D).

## DISCUSSION

A series of metabolic and enzymatic changes will occur in myocardial cells when myocardial ischemia makes the demand for oxygen and other metabolic substrates of myocardial cells greater than the supply, resulting in myocardial injury. After the restoration of blood flow in the myocardium with severe ischemia, the structure and function of cardiomyocytes will be further damaged, and sometimes even cause cell death, resulting in reperfusion injury ^[^^15^^]^. Meanwhile, myocardial I/R injury is the cause of cardiogenic death and disability ^[^^16^^]^. Therefore, the study of myocardial I/R injury and the mitigation of its damage to the heart plays an important role in reducing the mortality caused by heart disease and improving the quality of life of the population.

Studies have shown that miR plays an important role in myocardial I/R ^[^^17^^]^. MiR-346 is downregulated in I/R rat myocardial tissue, and the expression of miR-346 can target the miR-346/BAX signaling pathway to regulate the apoptosis level of rat myocardial tissue after I/R, thus alleviating the level of myocardial injury ^[^^18^^]^. In this study, BAX expression in I/R rat myocardial tissue was upregulated, and BAX expression level was downregulated after miR-124a inhibitor treatment, indicating that miR-124a downregulation could effectively alleviate the apoptosis of myocardial cells caused by I/R. Experiments in vivo and in vitro of Huang et al. showed that miR-374a-5p is downregulated in myocardial tissue. After overexpression of miR-374a-5p, myocardial injury level was significantly reduced, indicating that miR-374a-5p can be used as a potential therapeutic target for myocardial I/R ^[^^19^^]^. Downregulation of the expression level of miR-292-5p can alleviate myocardial I/R injury through the peroxisome proliferator-activated receptor (PPAR)-α/PPAR-γ pathway ^[^^20^^]^. In addition, the expression of miR-138 is downregulated in myocardial tissue after I/R, and overexpression of miR-138 can effectively reduce the myocardial infarction area and myocardial enzyme level caused by myocardial I/R, as well as inhibit the mitochondrial apoptosis caused by myocardial I/R ^[^^21^^]^.

Studies have shown that the upregulated expression of miR-208a after I/R injury further promotes cell injury and apoptosis, while the expression of cardiac enzymes such as LDH in H9c2 cells is upregulated ^[^^22^^]^, and miR-208a regulates Notch/NF-κB signaling pathway by targeting apoptosis-related factor CHD9, thus alleviating the level of I/R myocardial injury. In this study, the expression levels of NF-κB protein and myocardial enzymes LDH and CK in cardiac tissue of I/R rats were upregulated, and the expression levels of NF-κB protein and myocardial enzymes LDH and CK in cardiac tissue of I/R rats were downregulated after treatment with miR-124a inhibitor, indicating that inhibition of miR-124a could have a certain protective effect on myocardial injury after I/R.

Myocardial injury induced by cardiac I/R is closely related to intracellular calcium concentration. Cardiac I/R leads to increased concentration of Ca^2+^ in endothelial cells, which directly leads to injury to endothelial cells. It can also increase the expression of adhesion factors in endothelial cells, promote the adhesion of platelets and inflammatory cells to endothelial cells, and induce an inflammatory response in myocardium. Moreover, inflammation is considered to be the most important cause of tissue damage after organ ischemia. Studies have shown that the expression levels of inflammatory factors such as TNF-α, IL-6, and IL-1β in vivo are upregulated after I/R ^[^^23^^]^. Among them, TNF-α can activate and accumulate white blood cells in cardiomyocytes, enhance the phagocytosis of white blood cells, promote the adhesion between white blood cells and endothelial cells, and thus cause myocardial inflammation ^[^^24^^]^. IL-1 activates an inflammatory cascade that promotes TNF-α-mediated inflammation ^[^^25^^]^. Il-6 can induce the proliferation and differentiation of T cells and promote the differentiation of neutrophils ^[^^26^^]^.

In this study, ELISA results showed that the expressions of the inflammatory cytokines TNF-α, IL-6, and IL-1β in the myocardial tissue of I/R rats were significantly upregulated, so the myocardial cells showed a severe inflammatory response. At the same time, after the downregulation of miR-124a, the serum levels of the inflammatory factors TNF-α, IL-6, and IL-1β decreased, indicating that the downregulation of miR-124a can alleviate the cardiac injury caused by I/R to a certain extent. At the same time, pathological changes in rat myocardial tissue also showed that downregulation of miR-124a could effectively relieve the myocardial edema, red blood cell exudation, and myocardial fiber arrangement disorder caused by I/R, accompanied by inflammatory cell infiltration and local necrosis.

## CONCLUSION

In conclusion, inhibition of miR-124a by activation of Notch signaling pathway reduces myocardial enzyme content of LDH and CK, decreases myocardial infarction size and myocardial ultrastructure damage, reduces the expression of apoptosis-related proteins BAX and NF-κB, and reduces serum inflammatory factors TNF-α, IL-6, and IL-1β levels, playing a protective role in myocardial tissue after I/R. Therefore, miR-124a may be a potential therapeutic target for the treatment of I/R injury, and it is expected to be applied in biological and clinical applications.

**Table:** 

Authors' roles & responsibilities	
WX	Substantial contributions to the conception or design of the work; or the acquisition, analysis, or interpretation of data for the work; drafting the work or revising it critically for important intellectual content; agreement to be accountable for all aspects of the work in ensuring that questions related to the accuracy or integrity of any part of the work are appropriately investigated and resolved; final approval of the version to be published
SJ	Agreement to be accountable for all aspects of the work in ensuring that questions related to the accuracy or integrity of any part of the work are appropriately investigated and resolved; final approval of the version to be published
QL	Substantial contributions to the conception or design of the work; or the acquisition, analysis, or interpretation of data for the work; drafting the work or revising it critically for important intellectual content; agreement to be accountable for all aspects of the work in ensuring that questions related to the accuracy or integrity of any part of the work are appropriately investigated and resolved; final approval of the version to be published

## References

[1] Mokhtari-Zaer A, Marefati N, Atkin SL, Butler AE, Sahebkar A (2018). The protective role of curcumin in myocardial
ischemia-reperfusion injury. J Cell Physiol.

[2] High FA, Epstein JA (2008). The multifaceted role of notch in cardiac development and
disease. Nat Rev Genet.

[3] Schroeter EH, Kisslinger JA, Kopan R (1998). Notch-1 signalling requires ligand-induced proteolytic release of
intracellular domain. Nature.

[4] Artavanis-Tsakonas S, Rand MD, Lake RJ (1999). Notch signaling cell fate control and signal integration in
development. Science.

[5] Yekta S, Shih IH, Bartel DP (2004). MicroRNA-directed cleavage of HOXB8 mRNA. Science.

[6] Lee RC, Feinbaum RL, Ambros V (1993). The C elegans heterochronic gene lin-4 encodes small RNAs with
antisense complementarity to lin-14. Cell.

[7] Van Meter EN, Onyango JA, Teske KA (2020). A review of currently identified small molecule modulators of
microRNA function. Eur J Med Chem.

[8] Wang X, Xu H, Wang Y, Shen C, Ma L, Zhao C (2020). MicroRNA-124a contributes to glucocorticoid resistance in
acute-on-chronic liver failure by negatively regulating glucocorticoid
receptor alpha. Ann Hepatol.

[9] Fang QH, Shen QL, Li JJ, Yang Y, Guo JJ, Cheng Y (2019). Inhibition of microRNA-124a attenuates non-alcoholic fatty liver
disease through upregulation of adipose triglyceride lipase and the effect
of liraglutide intervention. Hepatol Res.

[10] Lu J, Ji H, Tang H, Xu Z microRNA-124a suppresses PHF19 over-expression.EZH2
hyper-activation.and aberrant cell proliferation in human
glioma (2018). Biochem Biophys Res. Commun.

[11] Sebastiani G, Po A, Miele E, Ventriglia G, Ceccarelli E, Bugliani M (2015). MicroRNA-124a is hyperexpressed in type 2 diabetic human
pancreatic islets and negatively regulates insulin secretion. Acta Diabetol.

[12] Diao X, Sun S (2017). PMicroRNA-124a regulates LPS-induced septic cardiac dysfunction
by targeting STX2. Biotechnol Lett.

[13] Wang R, Wang M, Zhou J, Ye T, Xie X, Ni D (2019). Shuxuening injection protects against myocardial
ischemia-reperfusion injury through reducing oxidative stress, inflammation
and thrombosis. Ann Transl Med.

[14] Livak KJ, Schmittgen TD (2001). Analysis of relative gene expression data using real-time
quantitative PCR and the 2(-delta delta C(T)) method. Methods.

[15] Eltzschig HK, Eckle T (2011). Ischemia and reperfusion--from mechanism to
translation. Nat Med.

[16] Roger VL, Go AS, Lloyd-Jones DM, Adams RJ, Berry JD, Brown TM (2011). Heart disease and stroke statistics--2011 update a report from
the American heart association. Circulation.

[17] Zhou Y, Chen Q, Lew KS, Richards AM, Wang P (2016). Discovery of potential therapeutic miRNA targets in cardiac
ischemia-reperfusion injury. J Cardiovasc Pharmacol Ther.

[18] Lv X, Lu P, Hu Y, Xu T miR-346 inhibited apoptosis against myocardial
ischemia-reperfusion injury via targeting bax in rats (2020). Drug Des Devel. Ther.

[19] Huang ZQ, Xu W, Wu JL, Lu X, Chen XM (2019). MicroRNA-374a protects against myocardial ischemia-reperfusion
injury in mice by targeting the MAPK6 pathway. Life Sci.

[20] Zhu ZD, Ye JY, Niu H, Ma YM, Fu XM, Xia ZH (2018). Effects of microRNA-292-5p on myocardial ischemia-reperfusion
injury through the peroxisome proliferator-activated receptor-a/- signaling
pathway. Gene Ther.

[21] Liu Y, Zou J, Liu X, Zhang Q (2019). MicroRNA-138 attenuates myocardial ischemia reperfusion injury
through inhibiting mitochondria-mediated apoptosis by targeting
HIF1-a. Exp Ther Med.

[22] Zhang S, Zhang R, Wu F, Li X (2018). MicroRNA-208a regulates H9c2 cells simulated ischemia-reperfusion
myocardial injury via targeting CHD9 through notch/NF-kappa B signal
pathways. Int Heart J.

[23] Lunsford KE, Baird BJ, Sempowski GD, Cardona DM, Li Z, Weinhold KJ (2013). Upregulation of IL-1ß, IL-6, and CCL-2 by a novel mouse
model of pancreatic ischemia-reperfusion injury. Transplantation.

[24] Cheng X, Liao YH, Li B, Yang YL, Zhang JY, Lu BJ (2005). Zhonghua Xin Xue Guan Bing Za Zhi.

[25] Nissen SE, Tuzcu EM, Libby P, Thompson PD, Ghali M, Garza D (2004). Effect of antihypertensive agents on cardiovascular events in
patients with coronary disease and normal blood pressure the CAMELOT study:
a randomized controlled trial. 26. JAMA.

[26] Boldizsár F, Berki T, Miseta A, Németh P (2002). Effect of hyperglycemia on the basal cytosolic free calcium
level, calcium signal and tyrosine-phosphorylation in human
T-cells. Immunol Lett.

